# 

**Published:** 1893-04-22

**Authors:** 


					I Nn TWOPENCE. M
SJ?3' Vo1- Xlv- -April 22nd, 1893. nil JMl
Q?neral Poat Office ?? a Newspaper far
^ aumsaion in the United Kingdom and Abroad.]
yUMMLLMLW
Per Annum, 8s. 6d., post free.
Monthly Farts, 6d.; post free, 8d.
Ditto per Annum, 8s., post free.
No. 343. Vol. XIV.] Saturday,
April 22nd, 1893.
[Twopence.

				

